# Asymptomatic and pre-symptomatic infection in Coronavirus Disease 2019 pandemic

**DOI:** 10.1515/mr-2021-0034

**Published:** 2022-02-24

**Authors:** Yutong Wang, Ke Zheng, Wenjing Gao, Jun Lv, Canqing Yu, Lan Wang, Zijun Wang, Bo Wang, Chunxiao Liao, Liming Li

**Affiliations:** Department of Epidemiology and Biostatistics, School of Public Health, Peking University, Beijing, China; Peking University Centre for Public Health and Epidemic Preparedness and Response, Beijing, China; National Institute for Communicable Disease Control and Prevention, Chinese Center for Disease Control and Prevention, Beijing, China; Meinian Public Health Institute, Peking University Health Science Center, Beijing, China

**Keywords:** asymptomatic, coronavirus disease 2019, pre-symptomatic, severe acute respiratory syndrome coronavirus 2

## Abstract

With the presence of Coronavirus Disease 2019 (COVID-19) asymptomatic infections detected, their proportion, transmission potential, and other aspects such as immunity and related emerging challenges have attracted people’s attention. We have found that based on high-quality research, asymptomatic infections account for at least one-third of the total cases, whereas based on systematic review and meta-analysis, the proportion is about one-fifth. Evaluating the true transmission potential of asymptomatic cases is difficult but critical, since it may affect national policies in response to COVID-19. We have summarized the current evidence and found, compared with symptomatic cases, the transmission capacity of asymptomatic individuals is weaker, even though they have similar viral load and relatively short virus shedding duration. As the outbreak progresses, asymptomatic infections have also been found to develop long COVID-19. In addition, the role of asymptomatic infection in COVID-19 remains to be further revealed as the severe acute respiratory syndrome coronavirus 2 (SARS-CoV-2) variants continue to emerge. Nevertheless, as asymptomatic infections transmit the SARS-CoV-2 virus silently, they still pose a substantial threat to public health. Therefore, it is essential to conduct screening to obtain more knowledge about the asymptomatic infections and to detect them as soon as possible; meanwhile, management of them is also a key point in the fight against COVID-19 community transmission. The different management of asymptomatic infections in various countries are compared and the experience in China is displayed in detail.

Since the severe acute respiratory syndrome coronavirus 2 (SARS-CoV-2) was first discovered in December 2019, Coronavirus Disease 2019 (COVID-19) has caused a pandemic worldwide. As of 12 December 2021, more than 260 million cases have been confirmed globally, resulting in over 5 million deaths [1]. The clinical manifestations of COVID-19 range from severe pneumonia to mild acute upper respiratory symptoms, as well as asymptomatic carriers [2]. With the expansion of laboratory testing capabilities, more and more COVID-19 cases of asymptomatic and pre-symptomatic infection have been detected. They have drawn considerable attention to the COVID-19 pandemic. So far, there have been many studies on asymptomatic infections of COVID-19, but the results are hard to reach a consensus. Here we try to put current knowledge together to provide a relatively comprehensive understanding of asymptomatic infections in the COVID-19 pandemic. Firstly, the magnitude of asymptomatic cases is described to find out how common they are. And we explore the extent to which the asymptomatic carriers may play a role in the spread of SARS-CoV-2. We then discuss their immunity and how vaccination influences the asymptomatic infection. Recent perspectives into some emerging challenges of asymptomatic individuals, like long COVID-19 and new variants, are also described here. Ultimately, the different managements of asymptomatic infections in various countries are compared and the experience in China is displayed in detail. Due to the nature of asymptomatic infections, we still less understand their aspect of the COVID-19 spectrum. But this should not allow us to ignore their contributions to the COVID-19 pandemic. Not only is further research urgently needed to find out the truth about asymptomatic infections, but we should also emphasize the importance of screening and managing them.

## Definition of asymptomatic infection and pre-symptomatic infection in COVID-19

Asymptomatic infection can be defined as persons with laboratory-confirmed SARS-CoV-2 infection, who never develop COVID-19-related clinical symptoms such as fever, cough, or diarrhea during illness. And pre-symptomatic infection is defined as persons with laboratory-confirmed SARS-CoV-2 infection, who have no symptoms at diagnosis time or the early phase of infection but develop COVID-19-related clinical symptoms during follow-up [3].

## The magnitude of asymptomatic infections

### The proportion of asymptomatic infections estimated via testing

#### The estimated proportion of asymptomatic infections

Many viral infections such as influenza are associated with asymptomatic infections, with an estimated proportion of 5.2%–35.5% [4]. Asymptomatic infections had occurred during severe acute respiratory syndrome (SARS) and the Middle East respiratory syndrome (MERS), which were also caused by human coronaviruses (HCoVs), SARS-CoV (2003), and MERS-CoV (2012), respectively. In Taiwan, China, a survey of 623 healthy healthcare workers who treated SARS patients found asymptomatic seroconversions in only two hospitals where four out of 433 healthcare workers had SARS antibodies (0.92%) [5]. According to World Health Organization (WHO), of the 2,228 confirmed cases of MERS-CoV, 21% of cases were reported to be asymptomatic or mild symptoms in 2018 [6], and 49 of the 219 (22.4%) patients were reported as asymptomatic in 2019 [7]. Similarly, many studies have reported the proportion of asymptomatic SARS-CoV-2 among infections, which is calculated as the number of asymptomatic cases divided by the total number of COVID-19 infections. Of the 3,063 passengers on board the quarantined Diamond Princess Cruise ship who were extensively tested for SARS-CoV-2, 17.9% (95% confidence interval [CI], 15.5%–20.2%) of those who tested positive were asymptomatic [8]. Nishiura used the data from Japanese charter flights evacuated from Wuhan, China, to conduct reverse transcriptase-polymerase chain reaction (RT-PCR) detection on 565 Japanese. Among them, 13 tested positive for SARS-CoV-2, including four as asymptomatic at the time of testing. The estimated proportion of asymptomatic infection was 30.8% (95% CI, 7.7%–53.8%) [9].

So far, 14 reviews or meta-analyses, focusing on the proportion of asymptomatic infected persons, have been retrieved [2, 10], [11], [12], [13], [14], [15], [16], [17], [18], [19], [20], [21], [22]. The proportion of asymptomatic infections among all confirmed cases differs significantly in the available reports, which is as low as 1.2% and as high as 100%. The estimated proportion of asymptomatic infections reported in the earlier narrative reviews was 5%–96% [2, 10]. And the first systematic review (collecting studies published before April 2020) to estimate the proportion of asymptomatic patients found that studies with a sample size greater than 1,000 cases reported the proportion was 1.2%–12.9%, while studies that had a smaller sample size estimated a higher proportion up to 87.9% [11]. A meta-analysis [16] (collecting studies published before 30 April 2020) of 2,788 people infected with SARS-CoV-2, including 16 studies, showed that the proportion of asymptomatic infection was 48.2% (95% CI, 30%–67%) [12]. A systematic review (collecting studies published from 1 January 2020 to 13 May 2020) derived from 104 studies involving 20,152 cases showed that the proportion of asymptomatic individuals in COVID-19 patients was 13.34% (95% CI, 10.86%–16.29%), of which pre-symptomatic infection accounted for 7.64% (95% CI, 4.02%–14.04%) [13]. Also, a systematic review and meta-analysis (collecting studies published before 20 May 2020) of 41 studies with 50,155 confirmed cases of COVID-19 reported that the proportion of asymptomatic infections was 15.6% (95% CI, 10.1%–23.0%) [14]. With the limited medical resource in the early COVID-19 pandemic, testing was mostly restricted to severe cases, and therefore few asymptomatic infections were reported [15]. Meanwhile, due to discrepancies in the understanding of “asymptomatic infection”, the estimated proportions of asymptomatic infection widely varied. In the early studies, some research included pre-symptomatic cases inadvertently, which resulted in high percentages. As a study mentioned, among the 180 initially asymptomatic patients, 48.9% (95% CI, 31.6%–66.2%) were pre-symptomatic infection [14]. This implied nearly half of the patients who had no symptoms at the time of the test could be in the incubation period and would develop symptoms later.

Then, for more valid estimates of the proportion of asymptomatic patients, a systematic review (collecting studies published from January 2020 to July 2020) only included studies with a follow-up period of at least 7 days, involving 21,708 people, and defined asymptomatic infections as patients who remained asymptomatic for the entire follow-up period. It found that the proportion of asymptomatic infection ranged from 4% in Korea [23] to 40% in Italy [24] and the United States [25], and estimated the overall asymptomatic infection percentage was 17% (95% CI, 14%–20%) [16]. Another meta-analysis (collecting studies published before 25 August 2020) which only included symptom-related follow-up studies reported the pooled proportion of asymptomatic infection was 23% (95% CI, 16%–30%) [17]. And a living systematic review showed that 20% (95% CI, 17%–25%) of people infected with SARS-CoV-2 had no symptoms at all during the entire period of infection, which was estimated from 79 studies across all kinds of study settings. Specifically, the study also estimated that pre-symptomatic infections account for 80% (95% CI, 75%–83%) of SARS-CoV-2 asymptomatic infections, which needed to be subtracted to get the true asymptomatic infections [18].

#### Key elements which influenced the estimated proportion

##### Study design

In fact, these findings imply that study designs play a key role in accurate estimates of the asymptomatic infection proportion. Cross-sectional studies can only assess people’s symptom profile at the time of testing, but cannot distinguish the truly asymptomatic infections from pre-symptomatic ones. Even in some follow-up studies, the observation period after testing was not long enough to ascertain whether the symptoms would appear subsequently, which might have resulted in the overestimation [15]. Therefore, more studies with an appropriate study design and a sufficient follow-up period are needed. With a more mature understanding of COVID-19, the latest systematic review and meta-analysis considered that the true proportion of asymptomatic infections should remain stable, regardless of the stage of the epidemic and extent of contact tracing, while current studies are context-specific, like mass testing once an outbreak happened, which may have an overrepresentation of cases experiencing symptoms. Thus, the researchers recommended removing index cases to correct representational bias that would lead to underestimation of the percentage of asymptomatic cases [15]. By excluding index cases, the pooled estimate of the asymptomatic percentage of over 350 studies was 35.1% (95% CI, 30.7%–39.9%), higher than that of previous meta-analyses.

##### Gender

In addition, there are still several factors related to the differences in the proportion of asymptomatic infection, for instance, gender. It has been recognized as an obvious gender difference that men suffered from more severe symptoms and higher mortality than women during the COVID-19 pandemic [26]. Some studies have highlighted females were more likely to be asymptomatic than males as well. A case series deriving data from Wuhan, China observed that more women (66.7%) were asymptomatic [27]. And a meta-analysis reported the pooled asymptomatic infection proportion was 55.5% in women higher than 44.5% in men [12]. Possible biological evidence suggested that SARS-CoV-2 entered epithelial cells via angiotensin-converting enzyme 2 (ACE2) receptor, facilitated by transmembrane serine protease 2 (TMPRSS2) [28]. One single-cell RNA-sequencing (RNA-seq) analysis suggested that Asian males tended to have a higher expression of ACE2 in the lung [29]. On the other hand, gender-based hormones also play an essential role here. A hypothesis about the protective effect of circulating ACE2 levels on women is proposed to illustrate the discrepancy of asymptomatic infections between males and females. Specifically, estrogens seem to regulate the expression and activity levels of ACE2, and then influence the viral entry [30]. The androgen sensitivity model in which the androgen receptor regulates the transcription of TMPRSS2 and thus probably facilitates SARS-CoV-2 virus-cell fusion is also applied to explain men’s higher possibility to experience severe symptoms [31]. Meanwhile, lifestyle factors such as washing hands less and smoking more among men may be another explanation for gender differences [32].

##### Age

Current studies have shown that children are considered more easily to be asymptomatic. In a meta-analysis, the proportion of asymptomatic infections in children was 27.7% (95% CI, 16.4%–42.7%), much higher than in all age groups (9.0%, 95% CI, 5.5%–14.6%) [14]. Similarly, another meta-analysis revealed that compared with adults (30.3%) and the elderly (16.9%), children (49.6%) had the largest proportion of asymptomatic cases. A statistically significant trend that the asymptomatic percentage declined with age increasing was also found in recent research [15]. Moreover, a systematic review that focused on studies in family clusters with children demonstrated that the percentage of asymptomatic infections for children (32.4%) was higher than for adults (13.3%). Even if they were infected with the same virus strain under the same circumstance, children had milder symptoms compared with their caregivers [33]. Factors including the lower number and affinity of ACE2 receptors, the developing immune system, and the repeated exposure to numerous coronaviruses make children more likely to be asymptomatic carriers rather than severe cases [11, 12, 33, 34]. And as mentioned before, the androgen sensitivity model related to TMPRSS2 also accounts for the distinction of asymptomatic infections between children and adults [19, 31].

##### Target population

The overall proportion of asymptomatic infection also depends on the target population with different characteristics. An Icelandic study screened high-risk residents and the general population for asymptomatic infections. It found that 7% of the 1,924 people targeted for testing and 43% of 10,797 population screened were asymptomatic [35]. Also, one meta-analysis showed that the proportion of asymptomatic infections in the general population was 20%–75% and in close contact was 8.2%–50%. The proportion of asymptomatic infections was 95% (95% CI, 45%–100%) in obstetric patients and 54% (95% CI, 42%–65%) in nursing home residents, of whom 59% (95% CI, 49%–68%) and 28% (95% CI, 13%–50%) remained asymptomatic during follow-up [20]. According to the latest systematic review, among the tested population, the pooled percentage of asymptomatic individuals was 0.25% (95% CI, 0.23%–0.27%). However, it was 40.50% (95% CI, 33.50%–47.50%) among the confirmed population, and it was higher in pregnant women, airline or cruise passengers, and nursing home staff or residents, which were 54.11% (95% CI, 39.16%–69.05%), 52.91% (95% CI, 36.08%–69.73%) and 47.53% (95% CI, 36.36%–58.70%), respectively [22]. With asymptomatic infections making outbreak tracking complicated, it is crucial to include populations with different characteristics in further research to obtain robust epidemiological evidence for a better understanding of asymptomatic infections. And it is also necessary to carry out screening and quarantine of high-risk populations such as air or cruise passengers to reduce the transmission of SARS-CoV-2.

##### Accuracy of diagnostic testing

The accuracy of diagnostic testing is critical when identifying the asymptomatic. Present gold standard diagnosis technique is real-time RT-PCR on nasopharyngeal swabs (NPS) or oropharyngeal swabs (OPS) [32]. A systematic review included 61 studies, 43 of which used NPS for RT-PCR and 18 of which used antibody testing. In 43 studies using RT-PCR, the proportion of asymptomatic infections ranged from 6.3 to 100%, with a median of 65.9% (Interquartile Range [IQR], 42.8%–87.0%). In the 18 studies using antibody testing, the proportion of asymptomatic infections was 21.7%–85.0%, with a median of 41.2% (IQR, 32.6%–48.1%) [21]. It seems that the proportion of asymptomatic infections tested by RT-PCR is reported to be higher, while the estimated seroprevalence of antibodies to SARS-CoV-2 is lower. This may be due to the fact that studies or reports based on PCR results, including only cross-sectional data, cannot distinguish between pre-symptomatic and asymptomatic SARS-CoV-2 infection, leading to an overestimation of the proportion of asymptomatic infections by PCR. But multiple factors can also cause false-negative results, such as inappropriate sampling, inadequate virus load, the incubation period, and the possible mutations to escape detection [36]. The reliability of RT-PCR has been doubted due to its false-negative results, which would underestimate the asymptomatic infections and contribute to the silent spread of SARS-CoV-2. In contrast, antibody-based studies were able to distinguish between pre-symptomatic and asymptomatic infections. Since the time of IgM or IgG antibody response is longer, serological testing is suitable to get the true number of asymptomatic infections in retrospective investigations [37, 38]. For instance, a study performed on healthcare workers in Germany showed that among 316 participants, four people who reported COVID-19 related symptoms had a negative PCR diagnosis, but their antibody testing was positive, and one subject who was asymptomatic only had a positive SARS-CoV-2 antibody detection. This finding is seen as a limitation of nasal swabs by the researchers since PCR diagnosis does not rule out the asymptomatic even though antibody is detected [39]. But serological testing still has limitations. For example, people cleared of SARS-CoV-2 infection by innate or mucosal immunization may be more likely to be asymptomatic, but would not be classified as asymptomatic infections in serosurvey, which may lead to an underestimation of the proportion of asymptomatic infections. In addition, serosurvey requires concurrent interviews or questionnaires about symptom status in blood samples. Participants were asked to accurately recall symptoms from weeks or even months earlier. In the context of the current COVID-19 pandemic, people may be more likely to notice and report symptoms that might otherwise be missed or ignored, resulting in lower estimates of the asymptomatic portion. It is found in a longitudinal PCR study in Wanzhou, China, the proportion of asymptomatic cases was 32.8% [40]. And in two nationally representative serosurveys conducted in England (n = 365, 104) and Spain (n = 61,075), the asymptomatic infections made up 32.4 and 33.0% of COVID-19 cases, respectively [41, 42]. The estimates were nearly identical. Consequently, to confirm the result and to accurately estimate the proportion of asymptomatic infections, the best way is to conduct large-scale longitudinal PCR tests using a representative sample of the national population [21].

### The proportion of asymptomatic infections estimated via model

A study based on 32,583 laboratory-confirmed cases [43], used modeling to reconstruct the full spectrum dynamics of COVID-19 in Wuhan from January 1 to March 8, 2020. The study found that 87% (53% lower bound) of infections before 8 March 2020 have not been identified (including asymptomatic and mild symptomatic infections). There were a large number of unascertained cases despite the high level of surveillance in Wuhan, indicating the presence of many asymptomatic infections [44]. A study demonstrated the significant impact of detecting and isolating asymptomatic infections using a modeling approach established in Argentina’s Mendoza province among 1.9 million people. According to the researchers, it could be applied to any other city or country. By setting Infection Fatality Rate (IFR) at ∼0.3% and Case fatality Rate (CFR) at ∼3%, the researcher concluded that asymptomatic infections accounted for 90% [45], consistent with the 86% of asymptomatic cases estimated by Li et al. [46]. The early estimate proportion of asymptomatic infections could be as high as 80% [47], but the researchers later revised it to 17%–20% [16, 18]. Studies reported that about 49% of people who were initially defined as asymptomatic developed symptomatic infections [14, 20].

Taken together, studies of asymptomatic infections have been limited by the heterogeneity of definitions, incomplete assessment of symptoms, and inadequate follow-up. Well-defined prospective longitudinal studies of asymptomatic infections, study designs that minimize selection and measurement bias, and serological tests combined with virological diagnostic methods can help better estimate the true proportion of asymptomatic infections [18].

## Transmissibility of asymptomatic infections

It is found that from 2002 to 2019, in SARS and MERS, the role of the asymptomatic transmission is negligible or not confirmed [48]. However, the SARS-CoV-2 transmission via asymptomatic infection has been proposed a lot [17].

The possibility of asymptomatic transmission of SARS-CoV-2 was first raised in a case report from China in which five COVID-19 patients were in contact with an asymptomatic family member who had traveled back from Wuhan. Based on the sequence of events, the traveler who remained asymptomatic during the whole 21-day follow-up period was presumed to have spread the infection to five other family members [49]. Also, in Hu et al.’s study, based on epidemiological investigation, one asymptomatic patient transmitted the COVID-19 virus to his family members, suggesting that asymptomatic infection can spread from person to person and is a source of COVID-19 [50].

Later, researchers not only confirmed the possibility of such transmission [50, 51] but also worked on quantifying the proportion, since the presence and extent of asymptomatic transmission could have a profound impact on the public health strategy. Specifically, if asymptomatic infections do play a major role in the spread of SARS-CoV-2, it may pose a greater risk to the disease control, so that conducting the mass screening is urgently required [52]. Otherwise, the large population-level testing could cause an unnecessary burden with considerable costs rather than reduce transmission [53, 54]. The keen debate regarding the extent to which asymptomatic infections contribute to disease transmission continues so far.

The transmissibility of an infectious disease depends on the infectivity of the pathogen [55], the susceptibility of the host population, the contact patterns between the exposed population and the infected population like close physical proximity and long duration of verbal interaction [56], and the environmental conditions involving temperature and humidity which could affect how long the virus remains infectious after exhalation. Here we particularly describe the infectivity of the SARS-CoV-2 virus in asymptomatic individuals, since it showed more different characteristics compared with symptomatic patients and there are limited data on how other factors affect the transmissibility of asymptomatic individuals.

### Infectivity of SARS-CoV-2 in asymptomatic infections

Measuring the true infectiousness of SARS-CoV-2 in asymptomatic individuals is complex. It is recognized that viral load dynamics and the duration of viral shedding are closely linked to SARS-CoV-2 transmission [57]. We also discussed the role of seroconversion in the infectivity of SARS-CoV-2 here.

#### Viral load

RT-PCR can recognize viral RNA but cannot determine the presence of the infectious virus, because the existence of RNA may be residual viral debris, not necessarily a live virus that can be transmitted [58, 59]. Virus culture is the only method to detect live viruses [60]. Wölfel et al. found that when the viral RNA load was below 5.40 Log_10_ RNA copies/mL, the success of isolating infectious virus was less than 5% [61]. Arons [62] cultured live virus from the respiratory tract of asymptomatic infected persons in one of three cases. Considering biosafety, stringent requirements on laboratories and professionals make cultivating live viruses unsuitable for routine infectivity testing [17, 57, 63]. Nevertheless, as virological evidence shows, infectivity can be inferred from the cycle threshold (Ct) value, which represents the number of PCR cycles required to detect SARS-CoV-2 RNA. And a lower value indicates a higher viral load and higher infectivity [61, 64, 65]. It was observed that SARS-CoV-2 cultures could not be obtained from samples of symptomatic patients with a Ct value higher than 34 [66], since a minimum amount of live virus was needed for onward transmission. A systematic review summarized that virus failed to propagate from samples of symptomatic cases equal to or above a Ct cutoff value which ranged from 24 to 35 [67]. Therefore, for a better understanding of the infectivity of asymptomatic cases, it is essential to pay attention to their viral RNA load.

The viral load of asymptomatic cases peaks at the early stage of illness [68], similar to the findings of symptomatic patients that viable virus was mainly isolated from respiratory tract samples within the first week of illness, and infected patients may have the strongest infectivity during the period, which provides a theoretical basis for effective transmission of SARS-CoV-2 [57]. However, the phenomenon in SARS-CoV-2 is different from that in SARS-CoV and MERS-CoV, where the viral load peaked 10–14 days and 7–10 days after illness, respectively [57].

What has been still controversial is the difference in viral load between asymptomatic and pre-symptomatic or symptomatic patients. Several studies reported that the Ct values were higher in asymptomatic patients than in pre-symptomatic and symptomatic patients (i.e., lower viral load) [68], [69], [70], [71], [72], [73], even during follow-up [74]. It was seen in the study conducted by Zhou et al. [68] that the viral load of asymptomatic patients was lower compared with pre-symptomatic patients in the incubation period. In a screening of 1,032 health care workers in the United Kingdom, Rivett et al. found that the viral load of 31 asymptomatic health care workers was significantly lower than that of the 30 symptomatic health care workers [71]. Another study showed a considerably high viral load in samples from fatal cases compared to asymptomatic infections [75].

But more studies suggest no difference in cycle thresholds detected by real-time RT-PCR assays or viral load between asymptomatic and symptomatic individuals [24, 62, 76], [77], [78], [79]. It was noted that in one of the earliest reports where asymptomatic cases remained symptom-free, the viral loads in samples of asymptomatic, pre-symptomatic, and symptomatic COVID-19 patients were similar [80]. And a systematic review and meta-analysis of 79 studies on SARS-CoV-2 virus dynamics and transmissibility found that symptomatic and asymptomatic participants had a similar viral load, but viral clearance was faster in asymptomatic infections, indicating a shorter period of asymptomatic infection but a similar potential transmission capacity to symptomatic infection at the outset [57], which is more accepted now [81]. This has also been found in the context of mutated COVID-19 infection. A study performed during the occurrence of the Alpha (B.1.1.7) variant demonstrated similar infectiousness among pre-symptomatic, asymptomatic, or mildly symptomatic (PAMS) and symptomatic individuals [82]. It is found that at the first positive test, the viral load of PAMS cases was only slightly lower than that of hospitalized patients; across the entire infection process, the viral load of hospitalized patients was slightly higher than that of non-hospitalized cases, while the viral load of the latter was slightly higher than that of PAMS cases. Based on the above evidence, it is almost beyond doubt that asymptomatic carriers are infectious [83]; however, with respect to viral load, whether the infectivity of asymptomatic cases is similar to that of symptomatic patients and whether the asymptomatic transmission could be a common route of SARS-CoV-2 transmissions, like the spread of symptomatic cases, are still questionable.

#### Virus shedding

Virus shedding, that is, virus particles are released from infected individuals during their daily actions, like breathing, eating, talking and so on, enhances the spread of infectious disease. Unlike patients with SARS [61], it turns out that virus shedding in COVID-19 symptomatic patients begins before they develop symptoms, reaches the highest levels right before or shortly after the onset of symptoms [84], and continues even beyond symptoms have been resolved [61]. From a longitudinal study conducted by Li et al., the virus shedding was observed in some asymptomatic patients likewise [51]. And the pattern of viral shedding observed in asymptomatic cases suggested that these individuals were infectious [85, 86].

Due to the high heterogeneity between studies, the estimate of the duration of virus shedding (interval from the first confirmed positive PCR result to the first negative conversion) varies widely. Nevertheless, most studies indicate that asymptomatic individuals shed infectious viruses faster than pre-symptomatic cases, and symptomatic patients have the longest duration of virus shedding [57]. Asymptomatic patients clear the virus faster and therefore are infectious for a shorter time [87], [88], [89]. A study conducted in Korea found that the median (SE) duration of viral shedding in asymptomatic patients was shorter compared with symptomatic (including pre-symptomatic) cases, which was 17 (1.07) days and 19.5 (0.63) days, separately (*p* = 0.07) [85]. And a study from Zhejiang in China found that the duration of virus shedding in pre-symptomatic patients was longer than in asymptomatic infections (48.0 vs. 24.0 days, *p* = 0.002) [69].

However, there are also different findings, like a study from Chongqing in China, where viral shedding duration in asymptomatic infection was significantly longer than that in symptomatic infection (19.0 vs. 14.0 days, *p* = 0.028) [90]. The reason for the discrepancy may be that in the early COVID-19 research, asymptomatic infections possibly included pre-symptomatic patients, who shedded the virus significantly longer than asymptomatic infections (seen from the baseline data of Chen et al.) [69]. And a study with three asymptomatic, six pre-symptomatic, and nine mildly symptomatic individuals reported that the median duration of virus shedding was 28.0, 11.5, and 31.0 days, respectively [51]. As the sample size of asymptomatic cases was too small, the result should be interpreted cautiously.

In addition, some studies reported no substantial difference between asymptomatic infections and symptomatic ones [68]. For instance, Zhang et al. identified that asymptomatic infections took longer to shed, though the difference was not significant (non-severe patients: 10.0 days; severe patients: 14.0 days; asymptomatic cases: 18.0 days) [91]. Further high-quality studies with a larger sample size and clear reports across the entire follow-up observation are warranted for better understanding the true difference.

Interestingly, it is worth noting that the long-term intermittent shedding of viral RNA has been reported in asymptomatic patients [51]. The viral RNA was still detected in two asymptomatic children 50 days after admission and re-appeared in eight asymptomatic patients after discharge [73]. As these findings imply, the great variation in the virus clearance suggests that we should pay attention to understanding viral shedding dynamics for asymptomatic COVID-19 in further research and public policies [73].

Determining the actual transmission capacity of asymptomatic infections is inherently complicated. It is believed that a higher viral load is independently associated with a longer duration of virus shedding [73], but most studies have found that asymptomatic individuals shed the virus faster than symptomatic individuals, even if their viral loads are similar. This may be related to other factors, involving host factors, like age, comorbidities, immune response, and objective factors in sampling, like sample types, Ct threshold, time to collect the sample for PCR and so on [92].

#### Seroconversion

The issue of seroconversion is another consideration for a better understanding of the infectiousness of SARS-CoV-2 [93]. Seroconversion is the transition from a seronegative state to a seropositive state, indicating the occurrence of humoral immunity. Nowadays, it is still unclear about the relationship between seroconversion and infectivity of SARS-CoV-2 among asymptomatic patients.

Some researchers have connected neutralizing antibody response and shedding of the infectious virus; they found that there was a very strong association between them with an odds ratio of 0.01 (95% CI, 0.003–0.08; *p* < 0.001) for isolation of infectious SARS-CoV-2 virus after seroconversion [94]. On the other hand, some researchers also worked on the association between viral loads and seroconversion. Earlier antibody release was observed in patients with higher peak viral loads, whereas patients without seroconversion showed very low viral loads [95]. Therefore, it turns out that the intensity of virus replication affects the induction of adaptive humoral immune responses, which in turn contributes to the shedding of the virus [95]. Based on the above findings, there is a hypothesis that seronegative patients are considered more infectious than seropositive patients. However, a study in Singapore just found that the possibility of close contacts being infected did not rely on the serology status of the index case [96]. Moreover, prolonged viral shedding was showed despite seroconversion [97], implicating the possibility of an extended contagious period.

But it seems to be different in asymptomatic carriers. Chen and colleagues proposed that the lower viral RNA load and shorter duration of virus shedding in asymptomatic infections were more likely to be caused by their relatively stronger antiviral immunity in which innate immunity and adaptive cellular immunity played a major role, rather than by the neutralizing antibody which referred to humoral immunity. Because their neutralizing antibody, no matter which was IgG or IgM, was dropping more rapidly in asymptomatic cases than in symptomatic patients [69]. It is found that the neutralizing antibody titer of asymptomatic infection is lower than that of symptomatic people [98]. The proportion of positive neutralizing antibodies was higher in the symptomatic population than in the asymptomatic population [99]. To date, the kinetic changes of neutralizing antibodies and their role in transmission are not well understood, especially in asymptomatic infections with more limited knowledge. But what we can be sure of the seroconversion is that serological testing could provide greater utility in finding asymptomatic patients and then ease and suppress the SARS-CoV-2 transmission.

### The transmission risk of SARS-CoV-2 in asymptomatic infections

Although studies to quantify the relative contributions of asymptomatic individuals to SARS-CoV-2 transmission are limited, current evidence tends to believe that symptomatic and pre-symptomatic transmissibility is stronger than asymptomatic infection [16, 18, 100, 101].

#### Evidence from contact tracing

So far, plenty of the studies have utilized the data of detailed contact investigations to obtain the secondary attack rate (SAR). The secondary attack rate, calculated as the number of newly infected cases among susceptible contacts of primary cases divided by the total number of susceptible contacts, generally reflects the infectivity of a virus [102].

Several studies estimated that the SAR of asymptomatic infection may be 3–25 times lower than that of symptomatic individuals [18, 100, 103, 104]. One study showed that the SAR of symptomatic and asymptomatic patients was 4.1% (128/3,136, approximately 38% contribution from pre-symptomatic cases) and 1.1% (12/1,078), respectively, indicating that the risk of transmission in symptomatic cases was higher than that in asymptomatic cases (OR 3.79, 95% CI 2.06–6.95) [105]. One study included 3,790 close contacts of 628 index cases who completed quarantine between 1 August and 11 October 2020. Its results showed that the incidence of COVID-19 was 3.85 (95% CI 2.06–7.19; *p* < 0.0001) times higher in close contacts of symptomatic index cases than in those of asymptomatic index cases [96]. In a household-centered survey, the researchers reported that the risk of transmission in asymptomatic households was about one-fourth that of symptomatic infections [106]. According to a systematic review, the summary SAR in asymptomatic infected persons was lower than that of symptomatic infected persons (RR 0.35, 95% CI 0.10–1.27) [18]. Another systematic review showed that the RR of asymptomatic transmission was 42% lower than that of symptomatic transmission (RR 0.58; 95% CI 0.34–0.99, *p* = 0.047) [16]. One systematic review and meta-analysis which compared the SARs among asymptomatic, symptomatic and pre-symptomatic index cases revealed that asymptomatic transmission contributed less to secondary infections. The SAR with a prediction interval was 1% (0–10%), 6% (5%–38%), 7% (1%–40%), separately [100]. According to the results of SAR, the transmission capacity of asymptomatic individuals is indeed weaker than that of symptomatic individuals; even though from the results on the viral load and viral shedding duration, whether asymptomatic or symptomatic individuals have higher infectiousness remains questioned. We suppose one reason for the weaker transmission risk in asymptomatic carriers is that asymptomatic infected people do not cough or sneeze as much, which is a prominent symptom of COVID-19 and can cause far more virus particles to flow out than talking and breathing [107].

Additionally, one study showed that the clinical manifestations of infected contacts varied according to the type of contact with index cases, that is, secondary cases were more likely to develop symptoms when they were exposed to symptomatic index cases and be asymptomatic when they were exposed to asymptomatic index cases [105]. Chen et al. proposed that asymptomatic infected persons were more likely to transmit asymptomatic infections [108].

#### Evidence from mathematical modeling studies

Some other researchers also used a mathematical model to estimate the transmissibility of asymptomatic COVID-19, which may provide additional information. In a prospective study that collected data of close contacts of COVID-19 patients, Chen et al. found no substantial difference between the infection rates of the close contacts of confirmed cases and asymptomatic infections, which were 6.30% (126/2,001) and 4.11% (6/146), respectively [108]. However, He et al. interpreted the outcomes with the susceptible-exposed-infectious-removed (SEIR) model and came to a different conclusion that the relative transmissibility of asymptomatic individuals could be significantly less than that of the symptomatic individuals. Even though the difference of risk of transmission per contact (*ρ*) was not statistically significant [(126/2,001)/(6/146) =] 1.5 (95% CI, 0.7–3.4), it was assumed that the average infectious period (*γ* ^−1^) of symptomatic individuals may be longer than that of asymptomatic individuals, therefore, combined with the two effects, the symptomatic group had a higher reproduction number [109].

Due to the lack of understanding of differences between asymptomatic and pre-symptomatic infections at the time, early studies overestimated the proportion of asymptomatic infections, which may affect the estimates of asymptomatic transmission. For instance, a previous study simulated SARS-CoV-2 transmission dynamics during 10–23 January 2020 (the early stage of the outbreak before initiation of travel restrictions) in China and estimated that the transmission rate of asymptomatic infection (presumably involving mild symptomatic patients and unconfirmed symptomatic cases) per person was 55% (95% CI, 46%–62%) that of symptomatic infection [46].

However, a variety of mathematical models have emerged to explore the contribution of asymptomatic infections over time. Utilizing data from digital contact tracing assisted with a mobile phone application, Ferretti et al. quantified the contribution of asymptomatic SARS-CoV-2 transmission as 6% in the early stages of the COVID-19 epidemic in China [110]. With discrete and two-type branching process models, Nakajo and Nishiura analyzed the cluster data among university students in Japan and estimated that the relative reproduction number of asymptomatically infected cases was 0.19 (95% CI, 0.03–0.66) compared with symptomatic cases, whose reproduction number was estimated at 1.14 (95% CI, 0.61–2.09) [111]. A susceptible-exposed-asymptomatic-confirmed-unconfirmed symptomatic-hospitalized-removed (SEAIUHR) model using data from January 21 to February 26, 2020 in Henan, China estimated the transmissibility of asymptomatic cases was 10% that of symptomatic ones [112]. A decision analytical model using data from a meta-analysis [113] indicated that based on baseline assumptions, COVID-19 patients without symptoms at that time contributed to around 59% of all transmission, of which 35% were pre-symptomatic individuals and 24% were asymptomatic individuals [114].

Most modeling studies have shown that the infectiousness of asymptomatic carriers is weaker than that of symptomatic individuals, but we still need to be cautious about these specific results. Despite the numerous mathematical models, the considerable variation among studies is not negligible. Parameter settings, including incubation period, generation time, serial interval and so on, are mostly estimated according to epidemic data which provided timeline and tracing. Since the data is limited and not detailed, the estimates may have considerable uncertainty [115]. Likewise, the different proportions of asymptomatic infections in different models may also lead to heterogeneity [110]. Due to the lack of generalizability, it is hard to externally validate the findings, and the credibility of results from mathematical models remains unclear [18].

### The role of asymptomatic infections in community transmission

Studies on the SAR found that the infectivity of asymptomatic infected persons is lower than that of symptomatic infected persons [23, 77, 116], [117], [118], but it needs to be quantified more accurately since considerable uncertainty remains about the role of asymptomatic cases in SARS-CoV-2 transmission [18].

Researchers remain divided over whether asymptomatic infections are driving the pandemic. On the one hand, it is almost recognized that asymptomatic infections are infectious, but may be less infectious than symptomatic infections so that they are not a driver of the pandemic [107]. A screening of nearly 10 million people conducted between May 14 and June 1, 2020 in Wuhan found no viable SARS-CoV-2 virus was cultured in samples from asymptomatic cases and all their close contacts were traced and tested negative for the COVID-19, which implied that asymptomatic individuals in this screening were unlikely to be contagious [119]. The conclusion may be related to the context of the research, in which individuals were just released from the strict lockdown. At that time, due to the severity of the outbreak, Wuhan implemented stringent control measures, which may influence the results to some extent. The above factors may influence the results to some extent. In addition, a systematic review that estimated the extent of asymptomatic infections and the risk of transmission demonstrated that asymptomatic transmission was unlikely to be the main driver of cluster or community transmission [16], with the evidence of lower asymptomatic transmission rates.

On the other hand, some studies suggest that a large number of asymptomatic infections are invisible according to symptom surveillance and may have more contact with the public than symptomatic infections (be quarantined), which significantly contributes to sustained community transmission [46, 110]. It is apparent that asymptomatic individuals are unlikely to be detected unless they initiatively seek medical treatment, participate in the screening test, or are investigated when an outbreak occurs. Meanwhile, as they are free from symptoms, other susceptible people may not keep a social distance from them. Consequently, the chance of exposure to the SARS-CoV-2 virus increases, particularly in places where people gather, in other words, communities. Li et al. found that asymptomatic individuals took an important part in the SARS-CoV-2 illness spectrum, and as shown in their research, most infections were not detected in the first epidemic wave. They suggested continuing surveillance for asymptomatic and mild infections because asymptomatic infections could spread the virus, even though they may not spread well [99].

In the former view that there is no strong evidence that asymptomatic populations are a major driver of transmission, some researchers considered mass testing in schools, colleges, and communities can be suspended [60]. Large-scale screening and contact tracing could build a staggering burden for healthcare services and impose high costs on the government. The false-positive PCR results may bring repeated testing and unnecessary quarantining. In addition, there is another concern that individuals who have been exposed to SARS-CoV-2 may suffer social stigma. Thus, they call for more accurate identification and isolation of cases. Currently, Australia does not recommend extensive PCR testing for asymptomatic contacts or communities [93].

On the contrary, people who perceive the importance of asymptomatic transmission suggest implementing screening and control strategies for asymptomatic infected individuals. They believe that strategies to control symptomatic cases alone are not sufficient to curb the spread of SARS-CoV-2 [62, 78] while taking measures against asymptomatic infection have certain significance in preventing SARS-CoV-2 transmission. Gandhi et al. regard asymptomatic transmission as the “Achilles’ Heel” of COVID-19 control strategies [120]. The spread of asymptomatic infections underscores the need for extensive testing and thorough contact tracing to detect asymptomatic infections and break undetected chains of transmission. Through proactive detection of symptomatic patients and expanded testing of close contacts, a large number of asymptomatic infections can be detected and isolated early, reducing the risk of transmission [121]. Also, social distancing is needed to prevent the spread of droplets of asymptomatic infection. Close contacts need to be quarantined to prevent further transmission of infected persons during asymptomatic periods [18].

It is clearly demonstrated that many outbreaks have been triggered and fueled by asymptomatic or pre-symptomatic infections [82, 110, 122]. And we cannot decide which one plays a more major role in community transmission events now. As Yang et al. reported, from October 2020 till February 2021, four of five COVID-19 outbreaks in China were caused by asymptomatic infections, and another outbreak was caused by a pre-symptomatic case [123]. However, in some studies, infections without symptoms were deemed to cause the epidemic, while they were eventually confirmed as pre-symptomatic. Although they presented no symptoms at the time of testing, they developed symptoms later [62]. Accordingly, for ascertaining whether asymptomatic infections could trigger an epidemic, based on what is currently known about the natural history of COVID-19, it is important to wait about 14 days to determine whether they are truly asymptomatic [124], even if asymptomatic infections can be contagious [86, 125]. Measuring the true potential of asymptomatic transmission is complex indeed since it is hard to observe the entire disease process of asymptomatic infected individuals. But knowledge gaps should not discourage the efforts to identify and take containment managements to asymptomatic individuals [78, 89]. Asymptomatic infected people should continue to take measures to reduce the spread of the virus, such as social distancing, hand hygiene, and wearing masks.

## Immunology and vaccination in asymptomatic infection

### Immunology in asymptomatic infection

At present, there is no clear answer to the question of what determines asymptomatic or symptomatic presentation after SARS-CoV-2 infection. As mentioned before, possible contributing factors include age, virus dose, etc. But another very important factor is the difference in the immune response. And the key question posed by current researchers about the nature of the asymptomatic infection is to answer: is it just a very mild form of infection or is it a form of effective immune control to suppress symptoms? Immune responses to asymptomatic infection mainly include innate, adaptive, and vaccine-induced immune responses. The characteristics of the antibody response to asymptomatic infection have been described previously. In the following sections, innate immunity and T cell-mediated immunity are mainly described. A study evaluating immunization in 37 asymptomatic infected individuals found that asymptomatic individuals had a weaker immune response to SARS-CoV-2 infection [90], and showed lower levels of 18 pro-inflammatory and anti-inflammatory cytokines. The adaptive immune system responds to pathogens in an antigen-specific manner to produce protective immunity. It consists of three main lymphocyte types: B cells, CD4+ T cells, and CD8+ T cells. The current study suggests that adaptive immunity is strongly activated during asymptomatic infections, but certain characteristics of T cells may differ from those of symptomatic cases [81]. Both SARS-CoV-2-specific CD4+ and CD8+ T cells were associated with milder symptoms [126]. Asymptomatic or mild COVID-19 individuals have durable functional T-cell-mediated immunity in the absence of an antibody response [127]. Evidence is still needed on the quality, quantity and durability of protective immunity after asymptomatic infection.

### Effect of vaccination on asymptomatic infection

In addition, most COVID-19 vaccine efforts focus on the initiation of neutralizing antibodies, and the CD4+ T cell response is critical to the success of most vaccines. In this context, a study has shown that the effect of asymptomatic infection immunity on the vaccine response is similar to that of symptomatic infection [81]. Notwithstanding, it is still necessary to explore the actual effectiveness of vaccines against asymptomatic individuals. Previous studies have well described the association between vaccination and a lower incidence of symptomatic SARS-CoV-2 infections, but vaccine impact on asymptomatic infections remains unclear.

The majority of studies suggested that vaccination can prevent asymptomatic SARS-CoV-2 infection. Jones et al. evaluated the short-term impact of first-dose BNT162b2 vaccination (Pfizer-BioNTech) on the positive rate of testing, and found that asymptomatic infections were reduced by four times among medical staff ≥12 days post-vaccination compared with unvaccinated ones, from 26/3,252 (0.8%, Wilson’s interval 0.6%–1.2%) to 4/1989 (0.2%, Wilson’s interval 0.1%–0.5%) [128]. Then, some researchers began to evaluate the effectiveness of full vaccination and after the longer observation period through the asymptomatic screening pathway. A retrospective cohort study performed 48,333 preprocedural COVID-19 molecular screening tests on 39, 156 patients in the US healthcare system, who kept no COVID-19 related symptoms. The majority of vaccinated patients received the BNT162b2 mRNA vaccine. It is found that patients who had received ≥1 dose of vaccine had a lower risk of asymptomatic infection compared with patients who were not vaccinated (RR 0.44; 95% CI, 0.33–0.60; *p* < 0.0001) [129]. Comparing patients who received the first dose of vaccination for more than 10 days with unvaccinated patients, the RR of a positive COVID-19 molecular test was 0.28 (95% CI, 0.16–0.49; *p* < 0.0001). In addition, comparing the two doses with the unvaccinated test, the RR for a positive test was 0.27 (95% CI, 0.12–0.60; *p* = 0.001). And a retrospective cohort study conducted in Israel found that compared with no vaccination, fully vaccination (more than 7 days after the second dose) with BNT162b2 was associated with a significantly lower incidence of asymptomatic SARS-CoV-2 infection, where incidence rate was 67.0 and 11.3 per 100,000 person-days, respectively, thus the adjusted IRR was 0.14 (95% CI, 0.07–0.31), and the corresponding estimated vaccine effectiveness (1−IRR) was 86%. When it was more than 21 days after the second dose, the adjusted IRR of asymptomatic infections reached 0.06, that is, the estimated vaccine effectiveness was 94% [130]. Similarly, another Israeli study showed that two doses of BNT162b2 were effective against asymptomatic COVID-19 infection. Specifically, the effectiveness of ≥7 days after the second dose was estimated to be 92%, and ≥14 days after the second dose was 94% [131]. A prospective, multicenter, cohort study in England indicated that the BNT162b2 vaccine had a preventive effect on both symptomatic and asymptomatic individuals among healthcare workers [132].

Given that vaccination may reduce asymptomatic SARS-CoV-2 infection, it is reasonable to assume that vaccination can reduce transmission. Nevertheless, there is a lack of data from observational studies and clinical trials [133]. Singanayagam et al. found that for fully vaccinated individuals, the SAR in household contacts exposed to the delta variant was 25% (95% CI 18–33), compared to 38% (95% CI 24–53) in unvaccinated ones, which indicated that despite being vaccinated, household contacts were still at risk of infection. The vaccine effectiveness in preventing infection with delta variant, regardless of symptoms, was estimated to be 34% (bootstrap 95% CI 15–60). Meanwhile, the SAR in household contacts who were exposed to fully vaccinated index cases was similar to those who were exposed to unvaccinated index cases (for vaccination: 25% [95% CI 15–35] and for unvaccinated: 23% [95% CI 15–31]), which showed that fully vaccinated individuals with breakthrough infections can still effectively transmit the virus in household settings. In a word, regardless of symptoms, vaccination is not sufficient to prevent the spread of delta variants in household settings with long-term exposures [134].

For better understanding how vaccination influences the magnitude of asymptomatic infections and transmissibility of asymptomatic infections, more evidence is urgently needed to provide support, and it is also important to understand the vaccine-initiated immune response in asymptomatic infections.

## Long COVID-19 in asymptomatic infection

Another key question for asymptomatic infection is whether asymptomatic infection carries a risk of developing long COVID. As the COVID-19 pandemic progresses, there is evidence that a large number of COVID-19 patients develop prolonged multi-organ symptoms and complications following the initial stages of acute infection, known as long COVID [135]. Long COVID is defined as a protracted period of persistent, fluctuating symptoms including fatigue, shortness of breath, headache, cognitive impairment, cough, chest pain and muscle pain [81, 136]. The Office for National Statistics (ONS) estimates that 1.1 million people in the UK have self-reported symptoms lasting more than four weeks [137]. Studies have suggested that the occurrence of long COVID seems to be related to the severity of acute symptoms of COVID-19. The Real-time Assessment of Community Transmission (REACT) study in the UK found that older age, women, overweight and obese individuals, and people hospitalized with COVID-19 were at higher risk of developing long COVID-19 [138]. A study of 2.2 million COVID-19 cases from FAIR Health in the US estimated that 19% of long COVID-19 cases were caused by asymptomatic infections [139]. In one study of 1,407 participants, about 32% of long COVID cases were initially asymptomatic at the time of testing [140]. On the question of whether mild or asymptomatic COVID-19 may also lead to systemic immunosuppression and long COVID, studies have suggested that neutrophil dysfunction leads to long-term endotype of immunosuppression in mild or asymptomatic COVID-19 convalescents [141]. Patients who were asymptomatic or mildly infected with SARS-CoV-2 still showed significant increases in the biomarker group of inflammation and stress response 40 days after infection, suggesting that biochemical and inflammatory pathways in the body can perturb long after SARS-CoV-2 infection has subsided [142]. Long COVID caused by asymptomatic infections may pose a significant public health burden in the future. The management measures of asymptomatic infections and how to deal with the long COVID damage caused by them need to attract the attention of governments and regulatory authorities.

## Asymptomatic infection of new variants

During the COVID-19 pandemic, the world had to deal with new viral variants. Some scholars consider that 2021 is shaping up to be the year of COVID-19 variants [143]. As of December 2, 2021, there are currently five designated Variants of Concern (VOCs) according to WHO. WHO has assigned simple, easy to say and remember labels for key variants of SARS-CoV-2, the virus that causes COVID-19, using letters of the Greek alphabet [144]. They are the B.1.1.7 (also known as 20I/501Y.V1) variant (named as Alpha variant) discovered in London, the UK in September 2020; the 501Y.V2 variant (named as Beta variant) discovered in South Africa in May 2020; the 501Y.V3 variant (named as Gamma variant), discovered in Brazil in November 2020; the 478K.V1 (named as Delta variant), discovered in India in October 2020; and the B.1.1.529 (named as Omicron variant), discovered in multiple countries in November 2021. The Omicron variant is the most heavily mutated variant to date [145]. Studies have suggested that sequence variants in the genome of SARS-CoV-2 may affect the infectivity, transmission, and pathogenicity of the virus [20, 146, 147]. These variants have prompted governments in many countries to impose restrictions, and new variants are being discovered with increasing frequency. In response to the challenge posed by variations of the SARS-CoV-2 virus, the pandemic has ushered in an era of genomic surveillance, with scientists tracking changes to the virus’s genome at an unprecedented speed and scale. But on a global scale, the surveillance is uneven. At present, only a few studies have reported asymptomatic infection of the SARS-CoV-2 virus variant [20, 146, 147]. According to the European Centre for Disease Prevention and Control (ECDC), the total number of confirmed cases of the SARS-COV-2 omicron variant reached 59 as of 1 December 2021, and all cases were asymptomatic or mild [148]. And a senior health official in Botswana said that 16 of the total 19 cases of omicron coronavirus variant detected in the country were asymptomatic [149]. It has been reported that three of eight specimens of SARS-CoV-2 Alpha (B.1.1.7) variant from Minnesota residents were asymptomatic [150]. The Alpha variant is reported to be more transmissible than certain other SARS-CoV-2 lineages [151]. One study reported an asymptomatic traveler arriving in Italy on an indirect flight from Brazil tested positive for the SARS-CoV-2 Gamma variant in a screening nasopharyngeal swab sample [152]. In addition, current research evidence suggests that there may be a higher proportion of asymptomatic infections in Gamma, Delta and Omicron variants than in Alpha and Beta variants. A study comparing 134 variant samples to 126 control samples found that while Alpha and Beta COVID-19 variants are associated with the higher transmission, patients infected with these two variants of COVID-19 are less likely to be asymptomatic when compared to the control group [153]. A large screening study of asymptomatic infections from Brazilian found 161 positive cases with a prevalence of the Gamma variant of 9.1%, which increased to 42.9% after 2 weeks [154]. According to WHO, cases of Delta variant infection have been reported in vaccinated populations, and most of them were mild or asymptomatic [155]. Recent preliminary studies have stated that Omicron appears to be “milder” than the Delta variant. A South Africa study showed that people infected with Omicron are 80% less likely to be admitted to a hospital when compared with other VOCs. Patients with omicron were 70% less likely to experience severe disease than earlier Delta infections. However, among those hospitalized, the risk of severe disease didn’t differ from other variants [156]. Another Scottish study showed that Omicron is two-thirds less likely to result in hospitalization vs. Delta [157]. Omicron might not be as dangerous as Delta, but it could lead to a huge spike in the numbers, for which people need to keep their guard up. One study showed that omicron had a much higher asymptomatic carrier rate than other VOCs, and the high prevalence of asymptomatic infection may be a major factor in the widespread and rapid spread of the variant globally, even among populations at high risk of SARS-COV-2 infection. The study found that 2.6% asymptomatic carriage during the Beta and Delta outbreaks and subsequently rose to 16% during the Omicron period in persons living with HIV [158]. And there is insufficient evidence whether SARS-CoV-2 variants will lead to changes in vaccine protection in asymptomatic infections. One study showed that a two-dose regimen of the ChAdOx1 nCoV-19 vaccine did not show protection against mild-to-moderate and asymptomatic SARS-CoV-2 infection due to the Beta variant [159]. In addition, a total of 17 breakthrough infections were found in a vaccinated cohort and all were mild or asymptomatic. The study found no measurable difference between cases and controls in post-vaccination neutralizing antibody titers against the wild-type, Alpha, and Delta, and anti-spike antibody titers, while neutralizing titers against the variants were considerably lower than those against the wild-type [160]. For now, it seems necessary to get vaccinated regardless of whether infected with the new variant. Further studies are needed to elaborate on whether the proportion of asymptomatic infections, transmission rate, vaccine protection, and therapeutics change with the emergence of SARS-CoV-2 variants worldwide, to what extent they change, and whether there is a difference with symptomatic infections.

## Management of asymptomatic infections

### Management of asymptomatic infected persons in various countries

Countries have developed strategies to respond to the COVID-19 pandemic that are consistent with their epidemiological features, capacities, and values [121]. The various strategies can be divided into two kinds. One is a containment strategy, which has been adopted by some countries and regions in the Asia-Pacific region, except Japan, and the other is a mitigation strategy, which has been implemented in most of Europe, except Germany [161]. The measures of containment strategy include mass testing or screening regardless of symptoms, contact tracing based on digital technologies, as well as different isolation and treatment depending on the severity of patients and the burden of the health system. As reported, South Korea, Singapore, New Zealand, Mongolia, Vietnam and Hong Kong (Special Administrative Region) have successfully contained COVID-19 through identifying and managing cases and their close contacts [121]. To curb the spread of asymptomatic patients, South Korea has carried out early COVID-19 screening. The government has significantly expanded testing, setting up more than 600 screening sites for nucleic acid tests to detect cases as early as possible [162]. Asymptomatic patients and mild cases are isolated at residential treatment centers or homes [161]. Singapore also maximizes detection of suspected cases through a network of public prevention clinics to test both symptomatic and asymptomatic people [163]. Workers in specific industries are routinely tested weekly or fortnightly [96]. Similarly, Singapore has arranged asymptomatic or mild patients to private hospitals or community facilities to reduce the burden on public hospitals [164]. And in New Zealand, some regions have adopted sentinel community testing to identify asymptomatic cases [165]. In addition, there is an exception that Germany, a European country, also conducts large-scale testing [161].

Meanwhile, many countries such as Japan, England and Norway have taken different approaches to face the challenge of asymptomatic infection of COVID-19. At the start of the outbreak, Japan and some countries in Europe mainly reserved testing for patients with severe symptoms [161, 166]. Asymptomatic individuals or mild cases recuperated at lodging facilities or homes and were monitored through communication devices [166]. Japan adopted this approach mainly because of its overstretched capacity in the governmental public-health service [167], while Norway did not recommend extensive testing due to its low infection rate and high possibility of false-positive results. Therefore, its asymptomatic testing was limited to nursing home staff and residents and close contacts of confirmed infections [168]. However, with local and global situations changing and new scientific evidence discovered, testing criteria have evolved. Since August 2020, a new regulation has been introduced in Norway that all people who suspect they may be infected can be tested without an initial evaluation by a community doctor [169]. Spain also plans to expand COVID-19 testing to involve symptom-free cases with easing COVID-19 restrictions [161].

A study that compared strategies in six countries found that countries that conducted the containment strategies keep both numbers of newly confirmed cases per day and the mortality rate per 100,000 population lower than countries that implemented the mitigation strategies. Under South Korea’s containment strategies, although the epidemic curve was similar to that of the United Kingdom, France, and the United States, which had implemented mitigation strategies, the case fatality rate of South Korea was only about 1% of that in countries that have implemented mitigation measures [170].

In a word, the obvious difference between these two types of strategies lies in large-scale early screening. However, the relevant measures in China are still different from the containment strategy described above, that is, strict quarantine employed in China.

### Evaluating management through mathematical models

Mathematical models can not only depict the transmission dynamics of the COVID-19 pandemic but also play a key role in evaluating the effects of diverse intervention policies, especially for some non-pharmacological interventions. Several models show that the detection and the management of asymptomatic infections significantly diminish the effective reproduction number, the duration of lockdown, medical burden, and total fatality.

Under the same parameters as in Flaxman’s study (*R*_0_ = 14) [171] and assuming that the infectivity of asymptomatic individuals is half that of symptomatic patients, a SEIR model found that rapid and effective testing, combined with the isolation of asymptomatic infections, can control the epidemic. The effective reproduction number can be halved, when the efficiency of detection reaches 50% within 3 days of becoming infectious. If supplemented with other non-pharmaceutical interventions, the effective reproduction number can be reduced to less than 1. On the contrary, if asymptomatic infected persons are not isolated, the effective reproductive number will be greater than the *R*_0_ parameter and they will remain infectious until they recover [45]. And screening for asymptomatic infections can significantly reduce the time required for a complete lockdown. We can also see in the Mayorga study if 45% of asymptomatic infections can be found and isolated, the entire population does not need to be quarantined; meanwhile, the health care system would not collapse based on the ICU occupancy rate indicator [45].

The negative impact of the medical burden associated with the spread of asymptomatic infections was also confirmed in a mathematical model study of 14.8 million people in Bahia, Brazil. The research implied that considering the majority of the asymptomatic infections usually undetected, relaxing social distancing measures may impose an extra burden on the already deficient medical system [172].

### The experience of asymptomatic infection management has been proved to be effective in China

In the early pandemic of China, the evidence for asymptomatic infections was relatively lacking. The strategy for managing asymptomatic carriers was updated, step by step, based on the latest scientific knowledge that researchers have obtained. Table 1 shows the timeline of the early research and corresponding strategies adjustment. Asymptomatic SARS-CoV-2 infections attracted attention from a very early stage considering their potential population size and infectiousness. The first case report of the asymptomatic carrier was published on Jan 24, 2020 [173]; and four days later, on Jan 28, asymptomatic infections were suggested to be reported in the guideline (Version 3) for COVID-19 prevention and control in China. In the later weeks, evidence was further accumulated, including epidemiological evidence revealing that there was presumed asymptomatic carrier transmission and modeling evidence indicating that serial interval might be shorter than incubation period. On Feb 24, in the guideline (Version 5) for COVID-19 prevention and control, the close contacts of COVID-19 patients were defined as “exposure to a symptomatic case 2 days before symptom onset of the case” instead of “exposure to a symptomatic case after symptom onset” (or exposure to an asymptomatic case 2 days before the date on which the sample that led to confirmation was taken). In the next several weeks, continuous evidence indicated that the infectiousness of asymptomatic carriers might be similar to symptomatic cases from a virologic or epidemiological view. In response to the concerns of the population, from Mar 31, 2020, China started to release the number of new asymptomatic infections every day and those who go on to develop symptoms. On Apr 6, China COVID-19 Joint Prevention and Control Mechanism of the State Council released the Management Standards for Asymptomatic COVID-19 Cases.

**Table 1:** Timeline for early research on asymptomatic infections and strategy modified.

Date	Evidence	Country	Types of evidence	Source
Jan 24	Case report of asymptomatic infection	China	Case report	Lancet
Jan 28	[Strategy] Asymptomatic infections started to be reported			
Jan 30	A case of presumed asymptomatic carrier transmission	Germany	Epidemiological	JAMA
Feb 3	Serial interval shorter than the incubation period	Japan	Modeling	medRxiv
Feb 3	Estimation of the asymptomatic ratio	Japan	Modeling	medRxiv
Feb 17	Asymptomatic ratio in reported cases	China	Epidemiological	China CDC
Feb 19	Serial interval estimation	China	Modeling	medRxiv
Feb 19	Case report of asymptomatic infection	China	Case report	Lancet Infect Dis
Feb 20	Clinical characteristics of asymptomatic infections	China	Epidemiological/Virologic	medRxiv
Feb 21	A case of presumed asymptomatic carrier transmission	China	Epidemiological	JAMA
Feb 24	[Strategy] Close contact definition changed			
Mar 9	A case of presumed asymptomatic carrier transmission	China	Epidemiological	EID
Mar 12	The asymptomatic proportion of COVID-19	Diamond Princess cruise	Epidemiological	Euro Surveill
Mar 16	The asymptomatic proportion of COVID-19 in pediatric patients	China	Epidemiological	Pediatrics
Mar 19	The viral load detected in the asymptomatic patients was similar to that in the symptomatic patients	China	Virologic	N Engl J Med
Mar 26	No statistically significant difference between the infection rate in close contacts of symptomatic cases (6.30%) and that of asymptomatic infections (4.11%)	China	Epidemiological	Chin J Epidemiol
Mar 31	[Strategy] China started to release data on new asymptomatic infections every day			
Apr 1	Seven COVID-19 epidemiologic clusters in which pre-symptomatic transmission likely occurred were identified, and 10 such cases within these clusters accounted for 6.4% of the 157 locally acquired cases. Pre-symptomatic transmission occurred 1–3 days before symptom onset in the pre-symptomatic source patient	Singapore	Epidemiological	MMWR Morb Mortal Wkly Rep
Apr 6	[Strategy] *Management standards for asymptomatic COVID-19 cases* were released			

In the latest guideline (Version 8) for COVID-19 prevention and control published on May 11, 2021, the asymptomatic individuals were suggested to be detected mainly through nucleic acid testing of key populations such as close contacts, entry personnel, high-risk occupational groups, etc.; source of infection tracking; epidemiological investigation; population screening. Asymptomatic infections should be reported online within 2 h after confirmation of their testing results. Asymptomatic individuals would be isolated in designated places for 14 days until their PCR test changed to be negative twice with a sampling interval ≥24 h. During isolation, blood routine examination, computed tomography (CT) imaging, and antibody testing should be accomplished. When asymptomatic individuals met the diagnostic criteria of symptomatic cases, they would be transferred to designated hospitals for standard treatments. Contact tracing of asymptomatic carriers is a key strategy for identifying asymptomatic infections and reducing chains of transmission of SARS-CoV-2. In China, the close contacts of asymptomatic carriers 2 days (for those infected with Delta variant, 4 days) before their sample confirmation were managed in the same way as for close contacts of symptomatic cases. They would be 14-day quarantined in designated places, during which health surveillance and routine PCR testing would be implemented.

## Summary

This literature review provides a comprehensive description of asymptomatic infections in the COVID-19 pandemic, including their magnitude, transmissibility, and management, according to both real data and mathematical models. And we also put forward some emerging challenges and concerns to asymptomatic individuals, like long COVID and new virus variants.

The estimate of the proportion of asymptomatic individuals ranges widely in the early studies, which may be due to the small sample size, the characteristic of the target population, the method, and accuracy of diagnostic testing, as well as an ineligible issue, that is, limited knowledge of “asymptomatic infection”. The discrepancy of a definition of asymptomatic infection in most studies may lead to the mixing of pre-symptomatic and asymptomatic infections, resulting in the true proportion of asymptomatic infections being even lower than current estimates. Thus, New studies to measure the authentic proportion require robust methodologies, including ensuring adequate follow-up periods to distinguish between asymptomatic and pre-symptomatic cases. With the results of the systematic review and meta-analyses we retrieved, it seems that the true magnitude of asymptomatic individuals is between 17% and 23% [16], [17], [18]. However, from some high-quality evidence, like nationwide, representative serosurveys, it is believed that the proportion of asymptomatic infections is at least one-third.

More and more strong evidence confirms that asymptomatic infected persons are silently spreading the SARS-CoV-2 virus and may pose a threat to the world. The presence of asymptomatic cases reminds us whether they are also contagious like those who have symptoms. Determining the true transmission potential of asymptomatic carriers is critical, as it can help to decide if present strategies to control the COVID-19 pandemic are appropriate. Here we primarily discussed the infectivity of SARS-CoV-2 in asymptomatic individuals. Our findings suggest that there is no substantial difference in viral loads between symptomatic and asymptomatic persons infected with SARS-CoV-2; nevertheless, the duration of virus shedding among asymptomatic individuals appears to be shorter than symptomatic patients. Faster virus clearance means that asymptomatic cases remain infectious for a shorter time. However, due to the nature of asymptomatic infections, we are still unclear about when SARS-CoV-2 viral load peaks, and when the virus is no longer infectious, which can help us better understand the efficient spread of SARS-CoV-2 and has more profound consequences for current public health strategies. Furthermore, we wanted to find whether seroconversion could provide some information about the infectiousness of SARS-CoV-2 among asymptomatic infections, like among those who have symptoms. However, the current evidence is limited, so further research is needed.

We then tried to pull current knowledge together and quantify the relative contributions of asymptomatic individuals to the spread of SARS-CoV-2. Based on detailed contact investigations, symptomatic and pre-symptomatic infections are observed to have higher SARs than asymptomatic carriers. Similarly, several mathematical modeling studies also demonstrate that the asymptomatic transmission contributes less to the spread of COVID-19, compared with the symptomatic and pre-symptomatic transmission. This finding is consistent with the results of studies on viral loads and the duration of viral shedding.

Owing to the apparent symptoms manifested in symptomatic patients, they are more likely to be found and measures are more easily taken to control the spread caused by symptomatic infection. Thus, even though the transmission capacity of asymptomatic infected persons seems to be weaker, the role of them in community transmission is keenly debated. Many COVID-19 outbreaks have been discovered to be triggered and fueled by asymptomatic or pre-symptomatic infections. But we cannot decide whether pre-symptomatic or asymptomatic infections are the major trigger of COVID-19 outbreaks. Some outbreaks thought to be caused by asymptomatic carriers were ultimately identified as caused by pre-symptomatic individuals [62]. In a living systematic review, Qiu et al. considered pre-symptomatic infections rather than asymptomatic individuals more likely to be a trigger in some outbreaks [100]. It can be illustrated that this assumption is closer to the results of virus dynamics as described above. Additionally, it is found in symptomatic cases that the peak of viral loads and the highest level of virus shedding are detected right before or onset of the symptoms up to the first week of illness [84], which indicates that the highest infectiousness may occur at the early stage of the illness referring to pre-symptomatic phase [57]. Also, a study modeling the infectious profile of COVID-19 inferred that 44% (95% CI, 30%–57%) of secondary cases were infected in the pre-symptomatic stage of the index case [84]. Nonetheless, to date, the pre-symptomatic viral load peak has not been confirmed in any study from direct observations. Notably, in China, four of five COVID-19 outbreaks were reported to be caused by the asymptomatic case [123], and another was initiated by a pre-symptomatic case, from October 2020 till February 2021.

Under strict control management, the symptomatic transmission has been effectively contained. However, why there are more COVID-19 outbreaks in China caused by asymptomatic infections than by pre-symptomatic cases remains unsolved [123]. In fact, the transmissibility of an infectious disease is related to plenty of factors, such as the susceptibility of the host, the time and patterns of contact between the exposed and the infected people, environmental factors and even the immune status of the infected individuals which may affect the contagiousness of themselves. And the information about these factors in asymptomatic or pre-symptomatic transmissions is not well-known.

Despite the knowledge gap, we believe attaching importance to the role of those who are symptomless in the spread of SARS-CoV-2 is a key point to control the pandemic, since whether asymptomatic or pre-symptomatic infections could transmit the virus quietly. Compared with the prevention and control of the epidemic based on the data of symptomatic infected persons, it is found high-frequency nucleic acid testing for people who are asymptomatic infectious has improved the control of the COVID-19 pandemic. Nucleic acid sampling can detect the incidence of an epidemic, which is a key reference variable for epidemic prevention and control. Frequent sampling of asymptomatic infections will have three effects. (1) Frequent testing of the infectivity of asymptomatic infections will significantly improve the predictability of the pandemic; (2) Help make a more reasonable and timely epidemic prevention and control policy update in time; (3) The efficiency of new ways to reduce the spread of the epidemic can be evaluated in real-time [174]. We support the view of implementing screening and control strategies for asymptomatic infected individuals, but we also want to call attention to a few issues: more precise epidemiological investigation and contact tracing to reduce unnecessary testing and isolation; taking care of the privacy and mental health of individuals exposed to SARS-CoV-2 to avoid social stigma.

We described the different management of asymptomatic infected persons all over the world and especially introduced China’s management strategy, because the management of asymptomatic infections in China is more explicit, specific, and strict, according to the information available to us. However, since countries have adjusted their management strategies along with changes in the stage of the epidemic, our description may not be complete or updated.

In the context of the COVID-19 pandemic, the asymptomatic infection has also been found to cause prolonged COVID. Given that asymptomatic infection accounts for a significant proportion of the epidemic, there is an urgent need for more research data to confirm whether the risk of long COVID caused by asymptomatic infection differs from symptomatic infection. In addition, asymptomatic infections have also been found to trigger innate and adaptive immune responses. On the one hand, asymptomatic infection induces immunity with a lower disease burden, and some researchers believe that it can be regarded as a natural supplement for SARS-CoV-2 immunity to enhance herd immunity [81]. Asymptomatic infections, on the other hand, cause a large number of untraceable transmissions and increase the potential risk of prolonged COVID. Furthermore, the role of asymptomatic infection in COVID-19 is still to be revealed as the SARS-CoV-2 variant continues to emerge, making the situation more severe. As the pandemic enters the era of genomic surveillance, more evidence is needed to focus on whether variant infections lead to a greater proportion of asymptomatic infections.

In summary, this review provides a comprehensive discussion of asymptomatic infections and highlights the role of asymptomatic infections in COVID-19. At present, we still do not know much about the characteristics of asymptomatic infections, and the COVID-19 situation is changing so rapidly. But what is not to be questioned is that to better control COVID-19, screening of high-risk groups such as close contacts is still necessary, and rigorous epidemiological investigations and laboratory tests will help identify asymptomatic infected persons. In the face of constantly updated information, scientists need to continue to pay attention to the characteristics of asymptomatic infections in the future.
